# Mapeamento do Potencial da Superfície Corporal durante a Despolarização Ventricular em Atletas com Intervalo PQ Prolongado após Exercício

**DOI:** 10.36660/abc.20230179

**Published:** 2024-02-16

**Authors:** Natalya I. Ivonina, Alexey G. Ivonin, Irina M. Roshchevskaya

**Affiliations:** 1 Komi Science Centre of the Ural Branch of the Russian Academy of Sciences Department of Comparative Cardiology Syktyvkar Federação Russa Department of Comparative Cardiology – Komi Science Centre of the Ural Branch of the Russian Academy of Sciences, Syktyvkar – Federação Russa

**Keywords:** Atletas, Bloqueio Atrioventricular, Ventrículos Cardíacos, Teste Ergométrico, Mapeamento do Potencial da Superfície Corporal

## Abstract

**Fundamento:**

O prolongamento do intervalo PQ, geralmente associado a um atraso na condução atrioventricular, pode estar relacionado a alterações na propagação do impulso intraventricular.

**Objetivo:**

Avaliar, por meio do mapeamento do potencial de superfície corporal (BSPM), o processo de despolarização ventricular em atletas com intervalos PQ prolongados em repouso e após o exercício.

**Métodos:**

O estudo incluiu 7 esquiadores cross-country com intervalo PQ superior a 200 ms (grupo PQ Prolongado) e 7 com intervalo PQ inferior a 200 ms (grupo PQ Normal). O BSPM de 64 derivações unipolares do tronco foi realizado antes (Pré-Ex) e após o teste ergométrico de bicicleta (Pós-Ex). Mapas equipotenciais da superfície corporal foram analisados durante a despolarização ventricular. O nível de significância foi de 5%.

**Resultados:**

Comparado com atletas com PQ Normal, o primeiro e o segundo períodos de posição estável dos potenciais cardíacos na superfície do tronco foram mais longos, e a formação da distribuição de potencial “sela” ocorreu mais tarde, no Pré-Ex, nos atletas com PQ Prolongado. No Pós-Ex, o grupo PQ Prolongado apresentou um encurtamento do primeiro e segundo períodos de distribuições de potencial estáveis e uma diminuição no tempo de aparecimento do fenômeno “sela” em relação ao Pré-Ex (para valores próximos aos do Normal -Grupo PQ). Além disso, no Pós-Ex, a primeira inversão das distribuições de potencial e a duração total da despolarização ventricular em atletas com PQ Prolongado diminuíram em comparação com o Pré-Ex e com valores semelhantes em atletas com PQ Normal. Em comparação com atletas com PQ Normal, a segunda inversão foi mais longa no Pré-Ex e Pós-Ex em atletas com PQ Prolongado.

**Conclusão:**

Atletas com PQ prolongado apresentaram diferenças significativas nas características temporais do BSPM durante a despolarização ventricular, tanto em repouso quanto após o exercício, em comparação com atletas com PQ normal.

**Figure:**
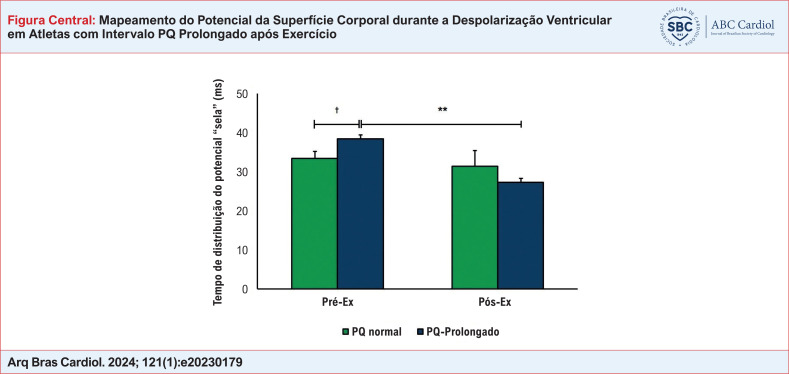


## Introdução

A participação regular intensiva em esportes resulta em alterações elétricas e estruturais no coração que podem se manifestar no eletrocardiograma (ECG) de superfície.^1^ Um intervalo PQ prolongado (mais de 200 ms) é apresentado nos ECGs dos atletas em 7,5 a 37,1% dos casos^2,3^ e refere-se a alterações comuns no ECG relacionadas ao treinamento.^4^ Em atletas, um intervalo PQ prolongado está associado a uma desaceleração na condução atrioventricular (AV) e é chamado de “bloqueio AV de primeiro grau”. Este atraso na condução do nó AV em atletas treinados pode ser consequência do aumento da atividade vagal ou de alterações intrínsecas no nó AV; diminui com o aumento da frequência cardíaca.^2,5^ A resolução do bloqueio AV de primeiro grau assintomático com exercício confirma sua origem funcional e exclui qualquer significado patológico.^4^

Em alguns casos, o prolongamento do intervalo PQ pode estar relacionado ao atraso na condução no sistema His-Purkinje.^6^ Os sinais de lentidão da condução intraventricular são o alargamento do complexo QRS e/ou aumento do tempo de pico da onda R (RWPT) no ECG nas derivações padrão.^7^ Ao mesmo tempo, verificou-se que a ativação dos ventrículos cardíacos começa antes mesmo do aparecimento do complexo QRS no ECG padrão.^8^ Portanto, um ECG de 12 derivações não é informativo o suficiente para estudar a fase inicial da despolarização ventricular, refletindo a propagação da excitação principalmente através do sistema de condução dos ventrículos cardíacos.

O mapeamento do potencial da superfície corporal (BSPM), baseado no registro síncrono de potenciais cardíacos de múltiplas derivações na superfície do tronco, é uma modalidade não invasiva para avaliar a atividade bioelétrica cardíaca e permite análises mais complexas e extensas do que as técnicas eletrocardiográficas padrão.^9,10^ As informações fornecidas pelo BSPM incluem os componentes espaciais, temporais e de amplitude do sinal de ECG e são usadas em ambientes experimentais e clínicos para detecção e diagnóstico de diversas condições patológicas.^11-13^ Anteriormente, a BSPM foi aplicada para investigar a ativação elétrica ventricular em pacientes com anormalidades na condução intraventricular.^14-17^ Estudos de despolarização ventricular por meio da BSPM no bloqueio AV de primeiro grau não foram realizados em atletas.

O objetivo do presente estudo foi fornecer a primeira caracterização detalhada do processo de despolarização ventricular em atletas com intervalo PQ prolongado em repouso e após o exercício usando BSPM.

## Métodos

### Participantes

O estudo envolveu pilotos de esqui cross-country do sexo masculino altamente treinados. Todos os participantes receberam uma explicação detalhada do estudo e o consentimento por escrito foi obtido de cada participante. Todos os participantes, no momento do estudo, não apresentavam doenças crônicas ou cardiovasculares e não faziam uso de medicamentos nem consumiam bebidas energéticas. Nenhum dos participantes se exercitou nas 24 horas anteriores aos procedimentos.

De acordo com os resultados da análise preliminar do ECG, dois grupos foram formados a partir dos participantes. O primeiro grupo (PQ Prolongado) incluiu atletas (n=7) com duração do intervalo PQ no ECG de repouso superior a 200 ms com ritmo sinusal estável sem alargamento do complexo QRS, característico do bloqueio AV de primeiro grau.^18^ O segundo grupo (Normal-PQ) consistiu em atletas (n=7) cujo intervalo PQ em estado de repouso não excedeu 200 ms, o que era típico para uma pessoa não atleta.^19^

### Ecocardiografia

A ecocardiografia bidimensional foi realizada com os indivíduos em repouso em decúbito lateral esquerdo, utilizando um scanner LOGIC P5 com transdutor de 5 MHz (General Electric Co, Waukesha, Wisconsin, EUA). As imagens cardíacas obtidas nos modos M e B na posição padrão paraesternal eixo longo e quatro câmaras, de acordo com as diretrizes da Sociedade Americana de Ecocardiografia,^20^ foram utilizadas para medir o diâmetro diastólico final do ventrículo esquerdo (DDVE), -diâmetro diastólico do ventrículo direito (DDVD), espessura da parede septal intraventricular (EPSI) e espessura da parede posterior do ventrículo esquerdo (EPVE).

### Registro e análise da atividade elétrica do coração

**Figura 1:**
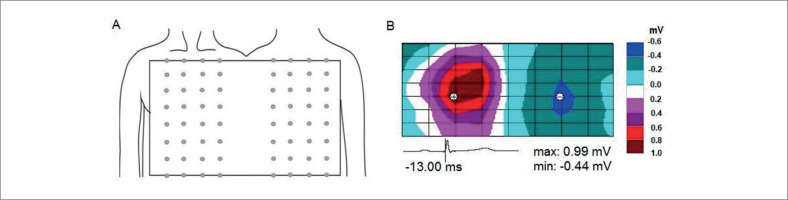
A) Posições dos eletrodos na superfície do tronco. B) Mapa equipotencial instantâneo da superfície corporal durante o complexo QRS. A área de potenciais positivos é de cor quente e a área de potenciais negativos é de cor fria. Os sinais “+” e “-” denotam a localização dos extremos positivos e negativos, respectivamente. Abaixo do mapa, são mostrados o ECG_II_ com um marcador de tempo (linha vertical), o tempo relativo ao pico da onda RII e as amplitudes dos extremos positivos (máx.) e negativos (mín.). À direita do mapa, a escala de cores é mostrada. A parte esquerda do mapa representa a superfície ventral do corpo e a direita representa a superfície dorsal do corpo.

Os indivíduos foram submetidos ao estudo quando estavam sentados. ECGs unipolares foram registrados a partir de 64 eletrodos espaçados uniformemente nas superfícies ventral e dorsal do tronco, desde as clavículas até a borda inferior do tórax (Figura 1A). Os eletrodos foram fixados em 8 fileiras, cada uma contendo 8 eletrodos. Simultaneamente aos ECGs unipolares da superfície do tronco, os ECGs foram registrados em derivações bipolares padrão dos membros, cujos eletrodos foram colocados nos pulsos e tornozelos. O terminal central de Wilson (uma média dos potenciais dos membros) foi utilizado como referência para derivações unipolares. Os dados foram adquiridos utilizando um sistema customizado para mapeamento eletrofisiológico (largura de banda de 0,05 – 1000 Hz, taxa de amostragem de 4000 Hz e precisão de 16 bits). A qualidade da visualização do sinal foi verificada antes do registro. Canais com níveis de ruído excessivos foram excluídos de análises posteriores. Os potenciais cardíacos foram registrados no estado inicial (em repouso) (Pré-Ex) e dentro de 1 minuto após a cessação do exercício (Pós-Ex).

Com base nos ECGs do tronco, foram construídos mapas equipotenciais da superfície corporal, refletindo a distribuição espacial dos potenciais cardíacos em qualquer momento do ciclo cardíaco em um padrão plano da superfície do tronco alinhado a um plano retangular (Figura 1B). Os mapas de potencial de superfície corporal (BSPMs) analisaram a localização espacial e as trajetórias de mudança das áreas e extremos de potenciais positivos e negativos na superfície do tórax durante a despolarização ventricular. Foram determinados o tempo de início e término da despolarização ventricular (de acordo com padrão típico de distribuição de potencial) e o tempo de início e término da primeira e segunda inversões de áreas e extremos. A inversão foi definida como uma mudança na posição mútua das áreas de potenciais positivos e negativos na superfície torácica. As características temporais dos BSPMs foram apresentadas em ms em relação ao pico da onda R_II_ (até o pico R_II_ – com sinal negativo). Posteriormente, foram calculadas a duração de ambas as inversões, a duração dos períodos de posição estável dos potenciais cardíacos na superfície corporal e a duração total da despolarização ventricular. O primeiro período de estabilidade da distribuição do potencial cardíaco foi desde o início da despolarização até o início da primeira inversão. O segundo período de estabilidade ocorreu após a conclusão da primeira inversão antes do início da segunda inversão. O terceiro período de estabilidade ocorreu após a conclusão da segunda inversão antes da conclusão da despolarização ventricular nos BSPMs. Além disso, determinamos o tempo desde o início da despolarização ventricular até a conclusão da primeira inversão dos potenciais cardíacos, que foi acompanhada pela formação da distribuição de potenciais em “sela”.^21^

No ECG_II_ foram analisadas as durações da onda P, R-R, PQ (PR), QRS (RS) e intervalo QT, e o intervalo QT corrigido (QTc) foi calculado pela fórmula de Bazett. Nas derivações unipolares do BSPM correspondentes à posição dos eletrodos V_1_ e V_5_ do ECG convencional, calculamos o RWPT, que foi medido desde o início do complexo QRS até o pico da onda R. Para cada participante, as características do ECG padrão e os BSPMs foram determinadas a partir de três a cinco batimentos no Pré-Ex e Pós-Ex.

### Protocolo de exercícios

Os participantes receberam um monitor de frequência cardíaca com cinta torácica (RS200, Polar Electro Oy, Kempele, Finlândia) e realizaram um teste de capacidade física de trabalho (PWC170) em uma bicicleta ergométrica EX 1 (Kettler GmbH, Ense-Parsit, Alemanha). Os sujeitos foram instruídos a manter uma cadência de pedalada constante entre 70 e 80 rpm durante todo o teste. Após uma carga de trabalho inicial de 1,5 W/kg, a carga de trabalho foi aumentada a cada 2 minutos com base na frequência cardíaca nos últimos 10 segundos. O teste foi concluído ao se aproximar de uma frequência cardíaca de 170 bpm.^22^

### Análise estatística

A análise estatística foi realizada utilizando o pacote de software Statistica (versão 10.0, StatSoft, Tulsa, OK, EUA). A normalidade contínua dos dados foi verificada por meio do teste de Shapiro-Wilk, que constatou que os dados estavam alocados dentro da curva Gaussiana. Portanto, as comparações intragrupos foram realizadas por meio de testes-t pareados, e as comparações intergrupos foram analisadas por meio de testes-t não pareados. O nível de significância foi estabelecido em *p* < 0,05. Os dados foram expressos como média ± desvio padrão (DP). O tamanho amostral de (n = 7) por grupo proporcionou poder de 90% com nível de significância de 5% nos protocolos de cálculo do tempo de formação da distribuição de potencial “sela” nos BSPMs no Pré-Ex.

## Resultados

### Características do participante

As características clínicas basais foram comparáveis em ambos os grupos de atletas (Tabela 1).

**Tabela 1:** Características basais dos atletas

	PQ-Normal (n = 7)	PQ prolongado (n = 7)	p-valor
Idade (ano)	20,2±3,9	22,3±5,1	0,50
Altura (cm)	178,2±3,3	177,7±4,0	0,85
Massa corporal (kg)	74,2±7,3	76,0±4,6	0,71
ASC (m^2^)	2,0 ± 0,1	1,9±0,1	0,38
FC (bpm)	59 ± 4	63±12	0,31
DDVE (mm)	54,1±1,3	56,2±4,2	0,26
DDVD (mm)	26,2±2,9	27,3±4,0	0,63
EPSId (mm)	10,5±0,8	10,2±0,3	0,63
LVPWTd (mm)	9,9±0,7	9,2±0,3	0,11

Os dados são apresentados como média ± DP. PQ normal: atletas com intervalo PQ normal; PQ prolongado: atletas com intervalo PQ prolongado; ASC: superfície corporal; FC: frequência cardíaca; bpm: batimentos por minuto; DDVE: diâmetro diastólico final do ventrículo esquerdo; DDVD: diâmetro diastólico final do ventrículo direito; EPSId: espessura da parede do septo intraventricular na diástole; LVPWTd: espessura da parede posterior do ventrículo esquerdo na diástole.

### Parâmetros de ECG

Os parâmetros de ECG são apresentados na Tabela 2. A duração do intervalo PQ_II_ em atletas com PQ prolongado foi significativamente maior no Pré-Ex do que em indivíduos com PQ normal. Comparado ao Pré-Ex, o intervalo PQ_II_ foi menor no Pós-Ex em ambos os grupos. No Pós-Ex, o intervalo PQ_II_ em atletas com PQ prolongado permaneceram mais tempo do que em indivíduos com PQ normal. A duração dos intervalos R-R_II_ e QT_II_ nos atletas dos grupos estudados não diferiu no Pré-Ex. Comparados ao Pré-Ex, os Intervalos R-R_II_ e QT_II_ foram mais curtos no Pós-Ex em ambos os grupos. Não foram observadas diferenças intergrupo entre a duração dos intervalos R-R_II_ e QT_II_ no Pós-Ex. Não houve diferenças inter e intragrupos nas durações da Onda P_II_, QRS_II_, QTc_II_, RWPT_V1_ e RWPT_V5_.

**Tabela 2:** Parâmetros de ECG em atletas

	PQ-Normal (n = 7)		PQ prolongado (n = 7)	
	Pré-Ex	Pós-Ex	Pré-Ex	Pós-Ex
R-R_II_ (ms)	1028,3±60,8	540,4±72,5***	975,1±186,6	575,0±81,6**
Onda P_II_ (ms)	98,6±9,8	95,7±6,8	99,1±12,2	96,6±7,6
PQ_II_ (ms)	152,6±12,9	141,9±8,3*	208,1±4,7†††	160,4 ± 6,1***,†††
QRS_II_ (ms)	92,1±4,6	87,0±6,5	86,9±10,4	87,6±9,3
QT_II_ (ms)	413,1±24,8	295,6±25,6***	407,1±61,1	330,3±35,7*
QTc_II_ (ms)	407,8±25,6	403,6±20,9	413,7±40,6	436,6±38,2
RWPT_V1_ (ms)	28,3±6,5	24,1±3,3	26,0±6,9	24,3±3,1
RWPT_V5_ (ms)	32,3±4,5	29,6±4,3	32,6±4,1	34,0±4,6

Os dados são apresentados como média ± DP. PQ normal: atletas com intervalo PQ normal; PQ prolongado: atletas com intervalo PQ prolongado; Pré-Ex: estado inicial; Pós-Ex: 1 minuto a partir da cessação do exercício; RWPT: horário de pico da onda R.

* p < 0,05,

** p < 0,01,

*** p < 0,001 vs. Pré-Ex;

††† p< 0,001 vs. PQ normal ao mesmo tempo.

### Características espaciais do BSPM

**Figura 2:**
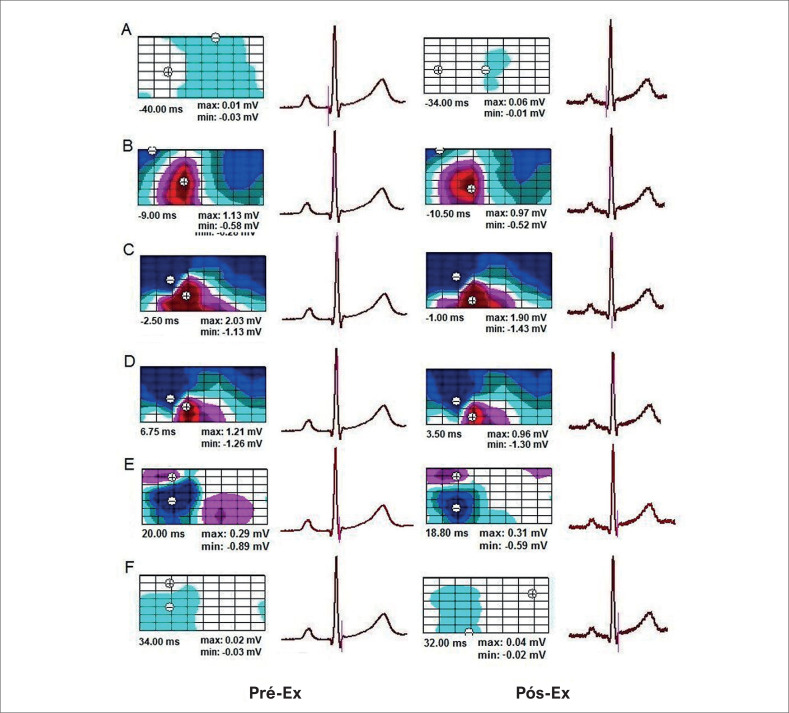
Mapas equipotenciais representativos da superfície corporal durante a despolarização ventricular no mesmo atleta com intervalo PQ prolongado. A) O início da despolarização ventricular. B) O início da primeira inversão das distribuições de potencial. C) A formação da distribuição de potencial “sela” (a conclusão da primeira inversão das distribuições de potencial). D) O início da segunda inversão das distribuições de potencial. E) A conclusão da segunda inversão das distribuições de potencial. F) A conclusão da despolarização ventricular. Pré-Ex: estado inicial; Pós-Ex: 1 minuto após o término do exercício. À direita de cada mapa, o ECG_II_ é mostrado com um marcador de tempo (linha vertical). As designações nos mapas são as mesmas da Figura 1.

No estado inicial, os padrões espaciais dos BSPMs durante a despolarização ventricular eram idênticos em ambos os grupos de atletas. A distribuição do potencial de superfície corporal, correspondente ao início da despolarização ventricular, foi observada antes do início da onda Q(R) no ECG_II_. Neste momento, o extremo positivo de pequena amplitude (0,01 – 0,03 mV) foi registrado na região da clavícula ou esterno, e o extremo negativo foi localizado no dorso (Figura 2A). Então, a área de potenciais cardíacos positivos expandiu-se por toda a superfície anterior, e os potenciais negativos – por toda a superfície posterior do tronco, enquanto as amplitudes dos extremos aumentaram. Durante a subida da onda R_II_, observamos a primeira inversão de distribuições de potencial– quando o máximo deslocou-se para a esquerda na parte frontal do tronco, e o mínimo desapareceu nas costas e apareceu sob a clavícula direita (Figura 2B). O movimento adicional do mínimo para baixo levou a um desvio da área negativa na forma de uma “sela” (Figura 2C). Após a formação do fenômeno de “sela” nos BSPMs, indicando a conclusão da primeira inversão dos potenciais cardíacos, seguiu-se um período de prazo de posição estável das áreas potenciais na superfície do tórax. Durante a descida da onda R_II_, a segunda inversão de distribuições de potencial foi observada (Figura 2D, E). A área positiva deslocou-se para as costas e por cima do ombro até a parte superior do tórax, e a área negativa espalhou-se por toda a parte frontal do tronco. A conclusão da segunda inversão das distribuições de potencial foi seguida por mais um período de posição estável dos potenciais cardíacos, que continuou até a conclusão da despolarização ventricular. Ao final da despolarização ventricular (onda S_II_ ascendente), o padrão espacial de distribuição de potencial não se alterou, enquanto as amplitudes dos extremos diminuíram (para 0,01 – 0,02 mV) (Figura 2F). Após o exercício, os padrões espaciais da BSPM durante a despolarização ventricular em atletas de ambos os grupos não se alteraram substancialmente em comparação com o estado inicial.

### Características temporais do BSPM

As características temporais do BSPM são apresentadas na Tabela 3. No Pré-Ex, o início da primeira e segunda inversões das distribuições de potencial em atletas com PQ Prolongado foi mais tardio do que em indivíduos com PQ Normal. Comparado ao Pré-Ex, o início da primeira inversão das distribuições de potencial foi posteriormente no Pós-Ex em atletas com PQ Normal. Em comparação com o Pré-Ex, um início mais tardio da despolarização ventricular, um início mais precoce e uma conclusão mais precoce da segunda inversão das distribuições de potencial foram revelados no Pós-Ex em atletas com PQ Prolongado. No Pós-Ex, os atletas com PQ Prolongado tiveram um início mais tardio da despolarização ventricular, um início mais tardio da primeira inversão, um início mais tardio e uma conclusão mais precoce da segunda inversão das distribuições de potencial em comparação com atletas com PQ Normal.

**Tabela 3:** Características temporais da BSPM em atletas

	PQ-Normal (n = 7)		PQ prolongado (n = 7)	
	Pré-Ex	Pós-Ex	Pré-Ex	Pós-Ex
Início da despolarização ventricular (ms)	–42,4 ± 4,6	–39,9±3,1	–44,8 ± 2,9	–34,7 ± 3,9**,†
Início da primeira inversão (ms)	–29,4 ± 3,6	–27,4±3,8***	–22,9 ± 5,8†	–20,2 ± 6,0†
Conclusão da primeira inversão (ms)	–9,0 ± 2,0	–8,5 ± 1,7	–6,4 ± 7,5	–7,4 ± 6,8
Início da segunda inversão (ms)	–2,0 ± 3,8	–2,6 ± 2,7	7,1±0,9†††	2,8 ± 2,2**,††
Conclusão da segunda inversão (ms)	19,4±4,4	18,4±2,9	19,4±2,5	13,8 ± 2,0**,††
Conclusão da despolarização ventricular (ms)	37,4±4,7	39,9±3,3	40,8±5,3	37,9±3,7

Os dados são apresentados como média ± DP. O tempo é mostrado em relação ao pico da onda RII. PQ normal: atletas com intervalo PQ normal; PQ prolongado: atletas com intervalo PQ prolongado; Pré-Ex: estado inicial; Pós-Ex: 1 minuto após o término do exercício.

** p < 0,01,

*** p < 0,001 vs. Pré-Ex;

† p < 0,05,

†† p < 0,01,

††† p < 0,001 vs. PQ normal ao mesmo tempo.

A duração da despolarização ventricular e suas fases individuais são apresentadas na Tabela 4. No Pré-Ex, dos atletas com PQ Prolongado, o primeiro e segundo períodos de distribuições de potencial cardíaco estáveis foram mais longos, e a segunda inversão de distribuições de potencial foi mais curta do que em Atletas com PQ normal. Em comparação ao Pré-Ex, não foram observadas alterações estatisticamente significativas na duração da despolarização ventricular e em suas fases individuais no Pós-Ex em indivíduos com PQ Normal. Comparada ao Pré-Ex, uma diminuição na duração do primeiro e segundo períodos das distribuições de potencial estáveis, bem como uma redução na duração da primeira inversão e a duração total da despolarização ventricular foram reveladas no Pós-Ex em atletas com PQ Prolongado. No Pós-Ex, a duração total da despolarização ventricular e a duração da primeira e segunda inversões em atletas com PQ Prolongado foram menores do que em Atletas com PQ normal.

**Tabela 4:** Durações da despolarização ventricular e suas fases individuais em atletas

	PQ-Normal (n = 7)		PQ prolongado (n = 7)	
	Pré-Ex	Pós-Ex	Pré-Ex	Pós-Ex
Despolarização ventricular (ms)	79,7±6,2	79,8±5,1	85,6±6,0	72,6 ± 5,7**,†
Primeiro período estável (ms)	12,9±3,3	12,5±2,6	21,9±5,3††	14,5±3,8**
Primeira inversão(ms)	20,4±3,2	18,9±3,4	16,5±7,9	12,8 ± 5,1*,†
Segundo período estável (ms)	7,0±3,6	5,9±3,2	13,5±7,2†	10,2±8,1**
Segunda inversão(ms)	21,4±6,6	21,1±4,1	12,2±2,9††	11,0±2,5†††
Terceiro período estável (ms)	18,0±4,3	21,4±1,9	21,4±5,4	24,1±2,8

Os dados são apresentados como média ± DP. PQ normal: atletas com normal intervalo PQ normal; PQ prolongado: atletas com intervalo PQ prolongado; Pré-Ex: estado inicial; Pós-Ex: 1 minuto após o término do exercício.

* p < 0,05,

** p < 0,01 vs. Pré-Ex.

† p < 0,05,

†† p < 0,01,

††† p < 0,001 vs. PQ normal ao mesmo tempo.

O tempo de formação da distribuição potencial “em forma de sela” nos atletas é apresentado na Figura Central. Comparada com Atletas com PQ normal, a formação da distribuição de potencial “sela” no Pré-Ex ocorreu posteriormente em atletas com PQ Prolongado (p < 0,05). No Pós-Ex, em Atletas com PQ normal, o tempo de formação do fenômeno “sela” nas BSPMs não se alterou significativamente. No Pós-Ex, em atletas com PQ Prolongado, a hora do aparecimento da “sela” diminuiu em comparação com Pré-Ex (p < 0,01) e não diferiu dos atletas com PQ normal.

## Discussão

No presente estudo, a atividade elétrica do coração foi avaliada durante a despolarização ventricular em atletas saudáveis, do sexo masculino, altamente treinados e com intervalo PQ prolongado em repouso e após exercício físico, utilizando eletrocardiografia convencional e BSPM.

No estado inicial, o intervalo PQ_II_ em atletas com PQ prolongado foi significativamente maior do que em atletas com PQ normal. De acordo com os dados,^5,23^ esses resultados podem ser principalmente devidos a uma desaceleração na condução AV em indivíduos com PQ prolongado. Após o teste de esforço, ambos atletas com PQ normal e atletas com PQ prolongado demonstraram uma diminuição na duração do PQ em relação ao estado inicial. O encurtamento do intervalo PQ_II_ após exercício em atletas com PQ Prolongado, segundo^4^ indicou a natureza funcional de seu alongamento em repouso e sugeriu o caráter benigno da lentidão da condução AV nesse grupo de indivíduos.

Anatomicamente, o local do atraso na condução AV que pode resultar em bloqueio de primeiro grau pode incluir os átrios.^6^ Nesses casos, a onda P no ECG será alargada.^24^ Em nosso estudo, no Pré-Ex, a duração da onda P_II_ não diferiu entre os grupos. Consequentemente, o prolongamento do intervalo PQ_II_ no ECG de repouso em atletas com PQ Prolongado não parecia ser devido a um atraso na condução atrial.

Um intervalo PR prolongado em combinação com um complexo QRS largo (mais de 120 ms) geralmente está associado a um atraso no sistema His-Purkinje.^6,24^ A condução intraventricular tardia também é caracterizada por um aumento do RWPT em derivações específicas do ECG.^7^ Neste estudo específico, as durações do complexo QRS_II_, RWPT_V1_ e RWPT_V5_ nos atletas dos grupos estudados em repouso não distinguiram e não excederam limites clínicos normais para adultos.^7,19^ Portanto, de acordo com o ECG padrão, o alongamento do intervalo PQ_II_ no grupo PQ Prolongado não foi acompanhado por condução lenta através dos ventrículos cardíacos. Para uma análise mais detalhada da despolarização ventricular nos atletas dos grupos comparados, utilizamos a abordagem BSPM.

Atualmente, os principais padrões de propagação do impulso elétrico através do sistema de condução cardíaca e ativação do miocárdio em funcionamento em humanos já foram identificados,^25-27^ a exibição desses processos na superfície do tórax também tem sido estudada.^8,28,29^ O início da ativação no ventrículo esquerdo ocorre sincronicamente em três regiões do subendocárdio, que crescem e, 15–20 ms após o início da ativação, se fundem em uma área. Então, a onda de ativação se espalha ao longo do septo interventricular em direção ao ápice do coração e se move através do tecido ventricular em direção endo-epicárdica.^25,26^ Na superfície do corpo, o início da ativação ventricular é refletido pelo aparecimento de uma pequena área positiva na região precordial e uma área negativa na escápula ou axila esquerda. Em seguida, o máximo se desloca para o lado esquerdo e para baixo, e o mínimo passa para o ombro direito, de onde desce para a região do apêndice xifoide. Nesse momento, a área negativa forma uma “sela”.^30,31^ O aparecimento do fenômeno “sela” nos BSPMs corresponde ao avanço da onda de excitação para o subepicárdio dos ventrículos.^25,28^ Em algumas pessoas, o aparecimento de “sela” coincide com a fase ascendente da onda R no ECG_II_, em outros – com seu pico.^29^ Após a formação da “sela”, nota-se uma localização estável das áreas de potenciais positivos e negativos, o que corresponde à despolarização da massa principal do miocárdio ventricular. Durante a descida da onda R_II_, a localização das áreas potenciais muda novamente – a área positiva moveu-se para as costas e parte superior do tórax e a área negativa espalhou-se por toda a parte anterior do tórax. Esse processo é explicado por uma mudança na direção da excitação através das paredes livres até as bases dos ventrículos. Até a conclusão da despolarização ventricular, o padrão espacial da distribuição de potencial da superfície corporal permanece quase inalterado,^8,31^ Neste estudo, as localizações das áreas e extremos positivos e negativos e sua dinâmica nas BSPMs durante a despolarização ventricular em atletas com intervalos PQ prolongados e normais no estado inicial foram semelhantes às de uma pessoa não atleta.

Foi demonstrado anteriormente que o exercício físico não leva a alterações nos padrões espaciais do BSPM durante o complexo QRS em uma pessoa saudável e destreinada.^32,33^ Após o teste ergométrico de bicicleta em atletas com PQ normal e PQ prolongado, a dinâmica das áreas e os extremos nas BSPMs durante a despolarização dos ventrículos não diferiram e foram típicos do estado de repouso. Isso permite aos autores do presente estudo concluírem que em atletas com intervalo PQ prolongado quando expostos ao exercício, os principais padrões de passagem da excitação pelo sistema de condução e funcionamento do miocárdio dos ventrículos permaneceram essencialmente inalterados.

Ao analisar os parâmetros temporais das BSPM nos atletas dos grupos estudados em repouso, não foram encontradas diferenças na duração global da despolarização ventricular. Porém, nos atletas do grupo PQ Prolongado em repouso, o primeiro e o segundo períodos de distribuição estável do potencial cardíaco foram mais longos do que nos atletas do grupo PQ Normal. Esses resultados sugeriram que a desaceleração da condução AV em atletas com PQ Prolongado em repouso foi acompanhada por um aumento na duração da excitação do septo interventricular e das camadas subendocárdicas do miocárdio ventricular (o primeiro período estável nos BSPMs), e excitação da massa principal dos ventrículos (o segundo período estável nas BSPMs). Ao mesmo tempo, o alongamento das duas fases de despolarização ventricular mencionadas acima no grupo PQ Prolongado foi compensado pelo encurtamento da segunda inversão da distribuição de potencial e, como resultado, a duração total da despolarização ventricular não diferiu entre os grupos. Após o exercício, os atletas do grupo PQ Prolongado apresentaram encurtamento do primeiro e segundo períodos da posição estável dos potenciais cardíacos na superfície corporal em relação ao estado inicial (para valores próximos aos dos atletas PQ Normal), o que poderia indicar o caráter funcional do prolongamento desses períodos de repouso. Além disso, após o exercício em atletas com PQ Prolongado, a duração da primeira inversão das distribuições de potencial e a duração global da despolarização ventricular diminuíram em comparação com o estado inicial e com os mesmos valores em atletas com PQ Normal. As causas exatas dessas mudanças não são claras e são necessárias mais pesquisas para estabelecê-las.

Estudos anteriores^16,17,21^ descreveram os padrões de BSPM durante a despolarização ventricular em pessoas com distúrbios de condução no sistema His-Purkinje. Em pacientes com bloqueio completo de ramo direito (BRD), o avanço da excitação no subepicárdio ventricular (que se reflete pela formação da distribuição de potencial “sela” nos BSPMs) é observado 44 ms após o início da ativação dos ventrículos cardíacos, em pacientes com BRD incompleto – 38 ms após o início da ativação ventricular.^21^ Neste estudo, em atletas do grupo PQ-Prolongado no estado inicial, foi observado o aparecimento do fenômeno “sela” em 38,4 ± 3,6 ms após início da despolarização ventricular. Em outras palavras, foi quase o mesmo que em pessoas com BRD incompleto,^21^ e significativamente mais tardio do que em uma pessoa saudável não treinada^29^ e em atletas do grupo PQ Normal em nosso estudo (33,4 ± 1,8 ms). Após o exercício, o tempo de aparecimento do fenômeno “sela” em atletas com PQ Prolongado diminuiu significativamente (para 27,3 ± 8,7 ms a partir do início da ativação ventricular) e não diferiu mais daquele em atletas com PQ Normal (31,4 ± 4,0 ms). Essa situação confirmou a origem funcional das alterações nas características temporais dos BSPMs durante a despolarização dos ventrículos em atletas com prolongamento do intervalo PQ em repouso.

### Limitações

A estimativa visual do movimento de áreas de potenciais positivos e negativos nos BSPMs, em vez de cálculos assistidos por computador, pode ser uma limitação. O número de atletas em nosso estudo foi relativamente pequeno. Uma população esportiva maior forneceria resultados mais precisos. O estudo incluiu atletas de resistência de alto nível (esquiadores cross-country) e os resultados podem diferir dependendo de qualquer especialização esportiva.

## Conclusão

Em resumo, atletas com intervalos PQ prolongados demonstraram diferenças essenciais nos parâmetros temporais dos BSPMs durante a despolarização ventricular em repouso e após o exercício, em comparação com atletas com valores normais de duração PQ. Esses achados podem contribuir para a compreensão do processo de remodelação elétrica em atletas.

## References

[1] Prakash K, Sharma S (2016). Interpretation of the Electrocardiogram in Athletes. Can J Cardiol.

[2] Drezner JA, Sharma S, Baggish A, Papadakis M, Wilson MG, Prutkin JM (2017). International Criteria for Electrocardiographic Interpretation in Athletes: Consensus Statement. Br J Sports Med.

[3] Fagard R (2003). Athlete's Heart. Heart.

[4] Corrado D, Pelliccia A, Heidbuchel H, Sharma S, Link M, Basso C (2010). Recommendations for Interpretation of 12-Lead Electrocardiogram in the Athlete. Eur Heart J.

[5] Huttin O, Selton-Suty C, Venner C, Vilain JB, Rochecongar P, Aliot E (2018). Electrocardiographic Patterns and Long-Term Training-Induced Time Changes in 2484 Elite Football Players. Arch Cardiovasc Dis.

[6] Schwartzman D, Zipes DP, Jalife J (2004). Cardiac Electrophysiology: From Cell to Bedside.

[7] Riera ARP, Abreu LC, Barros RB, Nikus KC, Baranchuk A (2016). R-Peak Time: An Electrocardiographic Parameter with Multiple Clinical Applications. Ann Noninvasive Electrocardiol.

[8] Roshchevskaya IM (2008). Cardioelectric Field of Warm-Blooded Animals and Man.

[9] Bond RR, Finlay DD, Nugent CD, Moore G, Guldenring D (2013). Methods for Presenting and Visualising Electrocardiographic Data: from Temporal Signals to Spatial Imaging. J Electrocardiol.

[10] Bergquist J, Rupp L, Zenger B, Brundage J, Busatto A, MacLeod RS (2021). Body Surface Potential Mapping: Contemporary Applications and Future Perspectives. Hearts.

[11] De Ambroggi L, Corlan AD (2007). Clinical use of Body Surface Potential Mapping in Cardiac Arrhythmias. Anadolu Kardiyol Derg.

[12] Franks MJ, Lawson L (2012). Body Surface Mapping Improves Diagnosis of Acute Myocardial Infarction in the Emergency Department. Adv Emerg Nurs J.

[13] Kania M, Maniewski R, Zaczek R, Kobylecka M, Fernandez HZ, Królicki L (2020). Optimal ECG Lead System for Exercise Assessment of Ischemic Heart Disease. J Cardiovasc Transl Res.

[14] Pastore CA, Tobias N, Samesima N, Martinelli M, Pedrosa A, Nishioka S (2006). Body Surface Potential Mapping Investigating the Ventricular Activation Patterns in the Cardiac Resynchronization of Patients with Left Bundle-Branch Block and Heart Failure. J Electrocardiol.

[15] Kittnar O, Riedlbauchová L, Adla T, Suchánek V, Tomis J, Ložek M (2018). Outcome of Resynchronization Therapy on Superficial and Endocardial Electrophysiological Findings. Physiol Res.

[16] Laszki-Szczachor K, Jagielski J, Rusiecki L, Sobieszczańska M, Janocha A (2006). Changes of Ventricular Activation Time in Patients with Left Anterior Fascicle Block and Bifascicular Block. Pol Arch Med Wewn.

[17] Samesima N, Pastore CA, Douglas RA, Martinelli MF, Pedrosa AA (2013). Improved Relationship Between Left and Right Ventricular Electrical Activation after Cardiac Resynchronization Therapy in Heart Failure Patients Can be Quantified by Body Surface Potential Mapping. Clinics.

[18] Sharma S, Drezner JA, Baggish A, Papadakis M, Wilson MG, Prutkin JM (2018). International Recommendations for Electrocardiographic Interpretation in Athletes. Eur Heart J.

[19] Macfarlane P, Lawrie T, Macfarlane P, van Oosterom A, Pahlm O, Kligfield P, Janse M, Camm J (2011). Comprehensive Electrocardiology.

[20] Lang RM, Bierig M, Devereux RB, Flachskampf FA, Foster E, Pellikka PA (2006). Recommendations for Chamber Quantification. Eur J Echocardiogr.

[21] Sugenoya J (1978). Interpretation of the Body Surface Isopotential Maps of Patients with Right Bundle Branch Block. Determination of the Region of the Delayed Activation within the Right Ventricle. Jpn Heart J.

[22] Ludyga S, Tränkner S, Gerber M, Pühse U (2021). Effects of Judo on Neurocognitive Indices of Response Inhibition in Preadolescent Children: a Randomized Controlled Trial. Med Sci Sports Exerc.

[23] Jacobson C (2008). Understanding Atrioventricular Blocks, Part I: First-Degree and Second-Degree Atrioventricular Blocks. AACN Adv Crit Care.

[24] Gorgels AP, Bar FW, Dulk KD, Wellens HH, Macfarlane PW, van Oosterom A, Pahlm O, Kligfield P, Janse M, Camm J (2011). Comprehensive Electrocardiology.

[25] Durrer D, van Dam RT, Freud GE, Janse MJ, Meijler FL, Arzbaecher RC (1970). Total Excitation of the Isolated Human Heart. Circulation.

[26] Ramanathan C, Jia P, Ghanem R, Ryu K, Rudy Y (2006). Activation and Repolarization of the Normal Human Heart Under Complete Physiological Conditions. Proc Natl Acad Sci USA.

[27] Opthof T, Remme CA, Jorge E, Noriega F, Wiegerinck RF, Tasiam A (2017). Cardiac Activation-Repolarization Patterns and Ion Channel Expression Mapping in Intact Isolated Normal Human Hearts. Heart Rhythm.

[28] Taccardi B, Punske BB, Lux RL, MacLeod RS, Ershler PR, Dustman TJ (1998). Useful Lessons from Body Surface Mapping. J Cardiovasc Electrophysiol.

[29] Mirvis D (1988). Body Surface Electrocardiographic Mapping.

[30] Medvegy M, Duray G, Pintér A, Préda I (2002). Body Surface Potential Mapping: Historical Background, Present Possibilities, Diagnostic Challenges. Ann Noninvasive Electrocardiol.

[31] De Ambroggi L, Corlan A, Macfarlane P, van Oosterom A, Pahlm O, Kligfield P, Janse M, Camm J (2011). Comprehensive Electrocardiology.

[32] Mirvis DM (1980). Body Surface Distribution of Exercise-Induced QRS Changes in Normal Subjects. Am J Cardiol.

[33] Takala P, Hänninen H, Montone J, Mäkijärvi M, Nenonen J, Oikarinen L (2001). Magnetocardiographic and Electrocardiographic Exercise Mapping in Healthy Subjects. Ann Biomed Eng.

