# Supplementary material for: Mapeamento do Potencial da Superfície Corporal durante a Despolarização Ventricular em Atletas com Intervalo PQ Prolongado após Exercício
Source: Arq Bras Cardiol. 2024 Feb 16;121(1):e20230179. [Article in Portuguese] doi: 10.36660/abc.20230179 (PMC11081108; doi:10.36660/abc.20230179)
Supplement: Translation

# Translation: Body Surface Potential Mapping during Ventricular Depolarization in Athletes with Prolonged PQ Interval after Exercise

**Authors:** Natalya I. Ivonina, Alexey G. Ivonin, Irina M. Roshchevskaya

**Affiliations:** 1 Syktyvkar Russian Federation Department of Comparative Cardiology – Komi Science Centre of the Ural Branch of the Russian Academy of Sciences, Syktyvkar – Russian Federation

**Keywords:** Athletes, Atrioventricular Block, Heart Ventricles, Exercise Test, Body Surface Potential Mapping

## Abstract

**Background:**

Prolongation of the PQ interval, generally associated with an atrioventricular conduction delay, may be related to changes in intraventricular impulse spreading.

**Objective:**

To assess, using body surface potential mapping (BSPM), the process of ventricular depolarization in athletes with prolonged PQ intervals at rest and after exercise.

**Methods:**

The study included 7 cross-country skiers with a PQ interval of more than 200 ms (Prolonged-PQ group) and 7 with a PQ interval of less than 200 ms (Normal-PQ group). The BSPM from 64 unipolar torso leads was performed before (Pre-Ex) and after the bicycle exercise test (Post-Ex). Body surface equipotential maps were analyzed during ventricular depolarization. The significance level was 5%.

**Results:**

Compared to Normal-PQ athletes, the first and second periods of the stable position of cardiac potentials on the torso surface were longer, and the formation of the “saddle” potential distribution occurred later, at Pre-Ex, in Prolonged-PQ athletes. At Post-Ex, the Prolonged-PQ group showed a shortening of the first and second periods of stable potential distributions and a decrease in appearance time of the “saddle” phenomenon relative to Pre-Ex (to the values near to those of the Normal-PQ group). Additionally, at Post-Ex, the first inversion of potential distributions and the total duration of ventricular depolarization in Prolonged-PQ athletes decreased compared to Pre-Ex and with similar values in Normal-PQ athletes. Compared to Normal-PQ athletes, the second inversion was longer at Pre-Ex and Post-Ex in Prolonged-PQ athletes.

**Conclusion:**

Prolonged-PQ athletes had significant differences in the temporal characteristics of BSPM during ventricular depolarization both at rest and after exercise as compared to Normal-PQ athletes.

## Introduction

Regular intensive participation in sports results in electrical and structural alterations within the heart that can manifest on the surface electrocardiogram (ECG).^1^ A prolonged (more than 200 ms) PQ interval is presented in athletes’ ECGs from 7.5 to 37.1% of cases^2,3^ and refers to common and training-related ECG changes.^4^ In athletes, a prolonged PQ interval is associated with a slowdown in atrioventricular (AV) conduction and is referred to as a “first-degree AV block”. This delay in AV nodal conduction in trained athletes may be a consequence of increased vagal activity or intrinsic changes in the AV node; it decreases with increasing heart rate.^2,5^ Resolution of asymptomatic first-degree AV block with exercise confirms its functional origin and excludes any pathological significance.^4^

In some cases, prolongation of the PQ interval may be related to delayed conduction in the His-Purkinje system.^6^ Signs of slowing intraventricular conduction are the widening of the QRS complex and/or an increase in the R-wave peak time (RWPT) on the ECG in standard leads.^7^ At the same time, it was found that the activation of the heart ventricles begins even before the appearance of the QRS complex on a standard ECG.^8^ Therefore, a 12-lead ECG is not informative enough to study the initial phase of ventricular depolarization, reflecting the propagation of excitation mainly through the conducting system of the heart ventricles.

Body surface potential mapping (BSPM), based on synchronous registration of cardiac potentials from multiple leads on the torso surface, is a non-invasive modality to assess cardiac bioelectric activity and allows more complex and extensive analysis than the standard electrocardiographic techniques.^9,10^ The information provided by BSPM includes the spatial, temporal, and amplitude components of the ECG signal and is used in both experimental and clinical settings for the detection and diagnosis of various pathological conditions.^11-13^ Previously, BSPM was applied in order to investigate the ventricular electrical activation in patients with intraventricular conduction abnormalities.^14-17^ Studies of ventricular depolarization by means of BSPM in first-degree AV block have not been conducted in athletes.

The purpose of the present study was to provide the first detailed characterization of the ventricular depolarization process in athletes with a prolonged PQ interval at rest and after exercise by using BSPM.

## Methods

### Participants

The study involved highly trained male cross-country ski racers. All participants received a detailed explanation of the study, and written consent was obtained from each participant. All participants, at the time of the study, did not have chronic or cardiovascular diseases and did not take medication or consume energy drinks. None of the participants exercised in the 24 hours before the procedures.

According to the results of the preliminary ECG analysis, two groups were formed from the participants. The first group (Prolonged-PQ) included athletes (n=7) with a PQ interval duration on the resting ECG of more than 200 ms with stable sinus rhythm without widening of the QRS complex, which is characteristic of first-degree AV block.^18^ The second group (Normal-PQ) consisted of athletes (n=7) whose resting state PQ interval did not exceed 200 ms, which was typical for a non-athlete person.^19^

### Echocardiography

Two-dimensional echocardiography was performed with the subjects resting in a left lateral decubitus position, using a LOGIC P5 scanner with a 5 MHz transducer (General Electric Co, Waukesha, Wisconsin, USA). The heart images obtained in M- and B-modes in the standard parasternal long-axis and four chambers position, according to the guidelines of the American Society of Echocardiography,^20^ were used to measure end-diastolic left ventricular diameter (LVEDD), end-diastolic right ventricular diameter (RVEDD), intraventricular septal wall thickness (IVST) and left ventricular posterior wall thickness (LVPWT).

### Registration and analysis of the electrical activity of the heart

Subjects underwent study when they were sitting. Unipolar ECGs were recorded from 64 electrodes evenly spaced on the ventral and dorsal surfaces of the torso from the collarbones to the lower edge of the chest (Figure 1A). The electrodes were attached to 8 rows, each containing 8 electrodes. Simultaneously with unipolar ECGs from the torso surface, ECGs were recorded in standard bipolar limb leads, the electrodes of which were placed on the wrists and ankles. The Wilson's central terminal (an average of the limb potentials) was used as a reference for unipolar leads. The data were acquired using a custom-designed system for electrophysiological mapping (bandwidth of 0.05 – 1000 Hz, sampling rate of 4000 Hz, and accuracy of 16 bits). The quality of the signal visualization was checked before registration. Channels with excessive noise levels were excluded from further analysis. Cardiac potentials were recorded in the initial state (at rest) (Pre-Ex) and within 1 minute after the cessation of exercise (Post-Ex).

**Figure 1:** A) Positions of electrodes on the torso surface. B) Instantaneous body surface equipotential map during QRS complex. The area of positive potentials is warm-colored, and the area of negative potentials is cold-colored. The signs “+” and “–” denote the location of the positive and negative extrema, respectively. Below the map, the ECG_II_ with a time marker (vertical line), the time relative to the R_II_-wave peak, and the amplitudes of the positive (max) and negative (min) extrema are shown. To the right of the map, the color scale is shown. The left part of the map represents the ventral body surface, and the right represents the dorsal body surface.

Based on torso ECGs, body surface equipotential maps were constructed, reflecting the spatial distribution of heart potentials at any given moment of the cardiac cycle on a flat pattern of the torso surface aligned to a rectangular plane (Figure 1B). Body surface potential maps (BSPMs) analyzed the spatial location and shifting trajectories of the areas and extrema of positive and negative potentials on the thorax surface during ventricular depolarization. The time of the onset and completion of the ventricular depolarization (according to typical potential distribution pattern) and the time of the beginning and completion of the first and second inversions of areas and extrema were determined. Inversion was defined as a change in the mutual position of the areas of positive and negative potentials on the thoracic surface. Temporal characteristics of the BSPMs were presented in ms relative to the peak of R_II_-wave (up to the R_II_-peak – with a minus sign). Subsequently, the duration of both inversions, the duration of the periods of the stable position of cardiac potentials on the body surface, and the total duration of ventricular depolarization were calculated. The first period of stability of the cardiac potential distribution was from the onset of the depolarization up to the beginning of the first inversion. The second period of stability was after the completion of the first inversion before the beginning of the second inversion. The third period of stability was after the completion of the second inversion before the completion of ventricular depolarization on the BSPMs. In addition, we determined the time from the onset of ventricular depolarization to the completion of the first inversion of cardiac potentials, which was accompanied by the formation of “saddle” potential distribution.^21^

On the ECG_II_, the durations of the P-wave, R-R, PQ (PR), QRS (RS), and QT intervals were analyzed, and the corrected QT interval (QTc) was calculated using Bazett's formula. In the unipolar BSPM leads corresponded to the position of the V_1_ and V_5_ electrodes of the conventional ECG, we calculated the RWPT, which was measured from the onset of the QRS complex to the R-wave peak. For each participant, characteristics of standard ECG and BSPMs were determined from the three to five beats at Pre-Ex and Post-Ex.

### Exercise protocol

The participants received a heart rate monitor with a chest belt (RS200, Polar Electro Oy, Kempele, Finland) and performed a physical work capacity test (PWC_170_) on a bicycle ergometer EX 1 (Kettler GmbH, Ense-Parsit, Germany). The subjects were instructed to maintain a constant pedaling cadence between 70 and 80 rpm throughout the test. After an initial workload of 1.5 W/kg, the workload was increased every 2 minutes based on the heart rate within the last 10 seconds of the actual stage. The test was completed upon approaching a heart rate of 170 bpm.^22^

### Statistical analysis

Statistical analysis was performed using a Statistica software package (Version 10.0, StatSoft, Tulsa, OK, USA). Continuous data normality was checked using the Shapiro-Wilk test, which found that the data were allocated within the Gaussian curve. Therefore, intragroup comparisons were performed by means of paired t-tests, and intergroup comparisons were analyzed by means of unpaired t-tests. The significance level was set at *p* < 0.05. Data were expressed as mean ± standard deviation (SD). The sample size of (n = 7) per group provided 90% power with a 5% significance level in protocols of calculating the time of formation of “saddle” potential distribution on BSPMs at Pre-Ex.

## Results

### Participant characteristics

Baseline clinical characteristics were comparable in both groups of athletes (Table 1).

**Table 1:** Baseline characteristics of athletes

	Normal-PQ (n = 7)	Prolonged-PQ (n = 7)	p-value
Age (year)	20.2±3.9	22.3±5.1	0.50
Height (cm)	178.2±3.3	177.7±4.0	0.85
Body mass (kg)	74.2±7.3	76.0±4.6	0.71
BSA (m^2^)	2.0 ± 0.1	1.9±0.1	0.38
HR (bpm)	59 ± 4	63±12	0.31
LVEDD (mm)	54.1±1.3	56.2±4.2	0.26
RVEDD (mm)	26.2±2.9	27.3±4.0	0.63
IVSTd (mm)	10.5±0.8	10.2±0.3	0.63
LVPWTd (mm)	9.9±0.7	9.2±0.3	0.11

Data are presented as mean ± SD. Normal-PQ: athletes with normal PQ interval; Prolonged-PQ: athletes with prolonged PQ interval; BSA: body surface area; HR: heart rate; bpm: beats per minute; LVEDD: left ventricular end-diastolic diameter; RVEDD: right ventricular end-diastolic diameter; IVSTd: intraventricular septal wall thickness at diastole; LVPWTd: left ventricular posterior wall thickness at diastole.

### ECG parameters

ECG parameters are presented in Table 2. The duration of the PQ_II_ interval in Prolonged-PQ athletes was significantly higher at Pre-Ex than in Normal-PQ individuals. Compared to Pre-Ex, the PQ_II_ interval was shorter at Post-Ex in both groups. At Post-Ex, the PQ_II_ interval in Prolonged-PQ athletes remained longer than in Normal-PQ athletes. The duration of the R-R_II_ and QT_II_ intervals in the athletes of the studied groups did not differ at Pre-Ex. Compared to Pre-Ex, the R-R_II_ and QT_II_ intervals were shorter at Post-Ex in both groups. No intergroup differences were observed between the duration of the intervals R-R_II_ and QT_II_ at Post-Ex. There were no inter- and intragroup differences in the durations of P_II_-wave, QRS_II_, QTc_II_, RWPT_V1_, and RWPT_V5_.

**Table 2:** ECG parameters in athletes

	Normal-PQ (n = 7)		Prolonged-PQ (n = 7)	
	Pre-Ex	Post-Ex	Pre-Ex	Post-Ex
R-R_II_ (ms)	1028.3±60.8	540.4±72.5***	975.1±186.6	575.0±81.6**
P_II_-wave (ms)	98.6±9.8	95.7±6.8	99.1±12.2	96.6±7.6
PQ_II_ (ms)	152.6±12.9	141.9±8.3*	208.1±4.7†††	160.4 ± 6.1***.†††
QRS_II_ (ms)	92.1±4.6	87.0±6.5	86.9±10.4	87.6±9.3
QT_II_ (ms)	413.1±24.8	295.6±25.6***	407.1±61.1	330.3±35.7*
QTc_II_ (ms)	407.8±25.6	403.6±20.9	413.7±40.6	436.6±38.2
RWPT_V1_ (ms)	28.3±6.5	24.1±3.3	26.0±6.9	24.3±3.1
RWPT_V5_ (ms)	32.3±4.5	29.6±4.3	32.6±4.1	34.0±4.6

Data are presented as mean ± SD. Normal-PQ: athletes with normal PQ interval; Prolonged-PQ: athletes with prolonged PQ interval; Pre-Ex: initial state; Post-Ex: 1 minute from the cessation of exercise; RWPT: R-wave peak time.

* p < 0.05,

** p < 0.01,

*** p < 0.001 vs. Pre-Ex;

††† p < 0.001 vs. Normal-PQ at the same time.

### Spatial BSPM characteristics

In the initial state, the spatial patterns of the BSPMs during ventricular depolarization were identical in both groups of athletes. The body surface potential distribution, corresponding to the beginning of ventricular depolarization, was observed before the onset of the Q(R)-wave on ECG_II_. At this time, the positive extremum of small amplitude (0.01 – 0.03 mV) was recorded in the region of the clavicle or sternum, and the negative extremum was located on the back (Figure 2A). Then, the area of positive cardiac potentials expanded over the entire anterior surface, and negative potentials – over the entire posterior surface of the torso, while the amplitudes of extrema increased. During the upslope of R_II_-wave, we observed the first inversion of potential distributions – when the maximum moved to the left down the front side of the torso, and the minimum disappeared on the back and appeared under the right collarbone (Figure 2B). Further movement of the minimum downward led to a deflection of the negative area in the form of a “saddle” (Figure 2C). After the formation of the “saddle” phenomenon on the BSPMs, indicating the completion of the first inversion of cardiac potentials, a period of stable position of potential areas on the thorax surface followed. During the downslope of R_II_-wave, the second inversion of potential distributions was observed (Figure 2D, E). The positive area moved to the back and over the shoulder to the upper chest, and the negative area spread over the entire front part of the torso. The completion of the second inversion of potential distributions was followed by a further period of stable position of cardiac potentials, which continued until the completion of ventricular depolarization. By the end of ventricular depolarization (upslope S_II_-wave), the spatial pattern of potential distribution did not alter, while the amplitudes of extrema decreased (to 0.01 – 0.02 mV) (Figure 2F). After exercise, the spatial BSPM patterns during ventricular depolarization in athletes of both groups did not change substantially in comparison with the initial state.

**Figure 2:** Representative body surface equipotential maps during ventricular depolarization in the same athlete with prolonged PQ interval. A) The onset of ventricular depolarization. B) The beginning of the first inversion of potential distributions. C) The formation of the «saddle» potential distribution (the completion of the first inversion of potential distributions). D) The beginning of the second inversion of potential distributions. E) The completion of the second inversion of potential distributions. F) The completion of ventricular depolarization. Pre-Ex: initial state; Post-Ex: 1 minute from cessation of exercise. To the right of each map, ECG_II_ is shown with a time marker (vertical line). The designations on the maps are the same as in Figure 1.

### Temporal BSPM characteristics

Temporal BSPM characteristics are presented in Table 3. At Pre-Ex, the beginning of the first and second inversions of potential distributions in Prolonged-PQ athletes was later than in Normal-PQ individuals. Compared to Pre-Ex, the beginning of the first inversion of potential distributions was later at Post-Ex in Normal-PQ athletes. Compared to Pre-Ex, a later onset of ventricular depolarization, an earlier beginning, and an earlier completion of the second inversion of potential distributions were revealed at Post-Ex in Prolonged-PQ athletes. At Post-Ex, Prolonged-PQ athletes had a later onset of ventricular depolarization, a later beginning of the first inversion, a later beginning, and an earlier completion of the second inversion of potential distributions compared to Normal-PQ athletes.

**Table 3:** Temporal BSPM characteristics in athletes

	Normal-PQ (n = 7)		Prolonged-PQ (n = 7)	
	Pre-Ex	Post-Ex	Pre-Ex	Post-Ex
Onset of ventricular depolarization (ms)	–42.4 ± 4.6	–39.9±3.1	–44.8 ± 2.9	–34.7 ± 3.9**.†
Beginning of the first inversion (ms)	–29.4 ± 3.6	–27.4±3.8***	–22.9 ± 5.8†	–20.2 ± 6.0†
Completion of the first inversion (ms)	–9.0 ± 2.0	–8.5 ± 1.7	–6.4 ± 7.5	–7.4 ± 6.8
Beginning of the second inversion (ms)	–2.0 ± 3.8	–2.6 ± 2.7	7.1±0.9†††	2.8 ± 2.2**.††
Completion of the second inversion (ms)	19.4±4.4	18.4±2.9	19.4±2.5	13.8 ± 2.0**.††
Completion of ventricular depolarization (ms)	37.4±4.7	39.9±3.3	40.8±5.3	37.9±3.7

Data are presented as mean ± SD. The time is shown relative to the R_II_-wave peak. Normal-PQ: athletes with normal PQ interval; Prolonged-PQ: athletes with prolonged PQ interval; Pre-Ex: initial state; Post-Ex: 1 minute from cessation of exercise.

** p < 0.01,

*** p < 0.001 vs. Pre-Ex;

† p < 0.05,

†† p < 0.01,

††† p < 0.001 vs. Normal-PQ at the same time.

The duration of ventricular depolarization and its individual phases are presented in Table 4. At Pre-Ex, of Prolonged-PQ athletes, the first and second periods of stable cardiac potential distributions were longer, and the second inversion of potential distributions was shorter than in Normal-PQ athletes. Compared to Pre-Ex, no statistically significant changes in the duration of ventricular depolarization and its individual phases were observed at Post-Ex in Normal-PQ individuals. Compared to Pre-Ex, a decrease in the duration of the first and second periods of the stable potential distributions, as well as a reduction in the duration of the first inversion and the total duration of ventricular depolarization, were revealed at Post-Ex in Prolonged-PQ athletes. At Post-Ex, the total duration of ventricular depolarization and the duration of the first and second inversions in Prolonged-PQ athletes were less than in Normal-PQ athletes.

**Table 4:** Durations of ventricular depolarization and its individual phases in athletes

	Normal-PQ (n = 7)		Prolonged-PQ (n = 7)	
	Pre-Ex	Post-Ex	Pre-Ex	Post-Ex
Ventricular depolarization (ms)	79.7±6.2	79.8±5.1	85.6±6.0	72.6 ± 5.7**.†
First stable period (ms)	12.9±3.3	12.5±2.6	21.9±5.3††	14.5±3.8**
First inversion (ms)	20.4±3.2	18.9±3.4	16.5±7.9	12.8 ± 5.1*.†
Second stable period (ms)	7.0±3.6	5.9±3.2	13.5±7.2†	10.2±8.1**
Second inversion (ms)	21.4±6.6	21.1±4.1	12.2±2.9††	11.0±2.5†††
Third stable period (ms)	18.0±4.3	21.4±1.9	21.4±5.4	24.1±2.8

Data are presented as mean ± SD. Normal-PQ: athletes with normal PQ interval; Prolonged-PQ: athletes with prolonged PQ interval; Pre-Ex: initial state; Post-Ex: 1 minute from cessation of exercise.

* p < 0.05,

** p < 0.01 vs. Pre-Ex.

† p < 0.05,

†† p < 0.01,

††† p < 0.001 vs. Normal-PQ at the same time.

The time of formation of “saddle-shaped” potential distribution in athletes is presented in Central Illustration. Compared to Normal-PQ athletes, the formation of the “saddle” potential distribution at Pre-Ex was later in Prolonged-PQ athletes (p < 0.05). At Post-Ex, in Normal-PQ athletes, the time of formation of the “saddle” phenomenon on BSPMs did not change significantly. At Post-Ex, in Prolonged-PQ athletes, the time of appearance of the “saddle” decreased compared to Pre-Ex (p < 0.01) and did not differ from that in Normal-PQ athletes.

## Discussion

In the present study, the electrical activity of the heart was evaluated during ventricular depolarization in healthy, highly trained male athletes with prolonged PQ interval at rest and after physical exercise by using conventional electrocardiography and BSPM.

In the initial state, the PQ_II_ interval in Prolonged-PQ athletes was significantly longer than in Normal-PQ athletes. In accordance with data,^5,23^ these results may be primarily due to a slowdown in AV conduction in Prolonged-PQ individuals. After exercise testing, both Normal-PQ and Prolonged-PQ athletes demonstrated a decrease in the PQ duration relative to the initial state. The PQ_II_ interval shortening after exercise in Prolonged-PQ athletes, according to,^4^ indicated the functional nature of its lengthening at rest and suggested the benign character of AV conduction slowing in this group of subjects.

Anatomically, the site of AV conduction delay that can result in first-degree block may include atria.^6^ In such cases, the P-wave on the ECG will be widened.^24^ In our study, at Pre-Ex, the P_II_-wave duration did not differ between the groups. Hence, prolongation of the PQ_II_ interval on the resting ECG in Prolonged-PQ athletes did not appear to be due to an atrial conduction delay.

A prolonged PR interval in combination with a wide QRS complex (more than 120 ms) is usually associated with a delay in the His-Purkinje system.^6,24^ Delayed intraventricular conduction is also characterized by an increase in the RWPT in specific ECG leads.^7^ In this particular study, the durations of the QRS_II_ complex, RWPT_V1,_ and RWPT_V5_ in the athletes of the studied groups at rest did not distinguish and did not exceed clinical normal limits for adults.^7,19^ Hence, according to the standard ECG, the elongation of the PQ_II_ interval in the Prolonged-PQ group was not accompanied by slowed conduction through the heart ventricles. For a more detailed analysis of ventricular depolarization in the athletes of the compared groups, we used the BSPM approach.

Nowadays, the main patterns of electrical impulse propagation through the cardiac conduction system and activation of the working myocardium in humans have been already identified,^25-27^ the display of these processes on the surface of the chest has been studied as well.^8,28,29^ The beginning of activation in the left ventricle occurs synchronously in three regions of the subendocardium, which grow and, 15–20 ms after the onset of activation, merge into one area. Then, the activation wave spreads along the interventricular septum towards the apex of the heart and moves through the ventricular tissue in an endo-epicardial direction.^25,26^ On the surface of the body, the beginning of ventricular activation is reflected by the appearance of a small positive area in the precordial region and a negative area in the scapula or left armpit. Then, the maximum shifts to the left side and lower, and the minimum passes to the right shoulder, from where it descends into the xiphoid process area. At that time, the negative area forms a “saddle”.^30,31^ The appearance of a “saddle” phenomenon on the BSPMs corresponds to the breakthrough of the excitation wave to the subepicardium of the ventricles.^25,28^ In some people, the “saddle” appearance coincides with the upslope phase of the R-wave on the ECG_II_, in others – with its peak.^29^ After the formation of the “saddle”, a stable location of the areas of positive and negative potentials is noted, which corresponds to the depolarization of the main mass of the ventricular myocardium. During the R_II_-wave downslope, the location of potential areas changes again – the positive area moved to the back and the upper chest and the negative area spread over the entire anterior chest. This process is explained by a change in the direction of the excitation through the free walls to the bases of the ventricles. Until the completion of ventricular depolarization, the spatial pattern of the body surface potential distribution remains almost unchanged.^8,31^ In this study, the locations of the positive and negative areas and extrema and their dynamics on BSPMs during ventricular depolarization in athletes with prolonged and normal PQ intervals in the initial state were similar to those in a non-athlete person.

It was previously shown that physical exercise does not lead to changes in the spatial BSPM patterns during the QRS complex in an untrained, healthy person.^32,33^ After the bicycle exercise test in Normal-PQ and Prolonged-PQ athletes, the dynamics of areas and extrema on BSPMs during depolarization of the ventricles did not differ and were typical for the resting state. This allows the authors of the present study to conclude that in athletes with a prolonged PQ interval when exposed to exercise, the main patterns of the passage of the excitation through the conducting system and working myocardium of the ventricles remained essentially unchanged.

When analyzing the temporal parameters of BSPMs in athletes of the studied groups at rest, no differences were found in the overall duration of ventricular depolarization. However, in the athletes of the Prolonged-PQ group at rest, the first and second periods of stable cardiac potential distributions were longer than in the athletes of the Normal-PQ group. These results suggested that the slowing down of AV conduction in Prolonged-PQ athletes at rest was accompanied by an increase in the duration of both excitation of the interventricular septum and subendocardial layers of the ventricular myocardium (the first stable period on BSPMs), and excitation of the main mass of ventricles (the second stable period on BSPMs). At the same time, the elongation of the two above-mentioned ventricular depolarization phases at the Prolonged-PQ group was compensated by the shortening of the second inversion of potential distribution, and as a result, the total duration of ventricular depolarization did not differ between the groups. After exercise, the athletes of the Prolonged-PQ group showed a shortening of the first and second periods of the stable position of cardiac potentials on the body surface relative to the initial state (to the values close to those in Normal-PQ athletes), which could indicate the functional character of the lengthening of these periods at rest. In addition, after exercise in Prolonged-PQ athletes, the duration of the first inversion of potential distributions and the overall duration of ventricular depolarization decreased compared to the initial state and with the same values in Normal-PQ athletes. The exact causes of these changes are unclear, and further research is needed to establish them.

Previous studies^16,17,21^ described the BSPM patterns during ventricular depolarization in persons with conduction disturbances in the His-Purkinje system. In patients with a complete right bundle branch block (RBBB), the breakthrough of the excitation on the ventricular subepicardium (which is reflected by the formation of the “saddle” potential distribution on the BSPMs) is noted at 44 ms after the onset of activation of the heart ventricles, in patients with incomplete RBBB – at 38 ms after the onset of ventricular activation.^21^ In this study, in athletes of the Prolonged-PQ group in the initial state, the appearance of the “saddle” phenomenon was observed at 38.4 ± 3.6 ms after the beginning of ventricular depolarization. In other words, it was almost the same as in people with incomplete RBBB,^21^ and significantly later than in an untrained healthy person^29^ and in athletes of the Normal-PQ group in our study (33.4 ± 1.8 ms). After exercise, the time of the “saddle” phenomenon appearance in Prolonged-PQ athletes significantly decreased (to 27.3 ± 8.7 ms from the onset of ventricular activation) and no longer differed from that in Normal-PQ athletes (31.4 ± 4.0 ms). This situation confirmed the functional origin of changes in the temporal characteristics of BSPMs during depolarization of the ventricles in athletes with prolongation of the PQ interval at rest.

### Limitations

Visual estimation of the movement of areas of positive and negative potentials on the BSPMs instead of computer-assisted calculations may be a limitation. The number of athletes in our study was relatively small. A larger sports population would provide more precise results. The study included top-level endurance athletes (cross-country skiers), and results may differ based on any sport specialization.

## Conclusion

In summary, athletes with prolonged PQ intervals demonstrated the essential differences in the temporal parameters of BSPMs during ventricular depolarization at rest and after exercise as compared to athletes with normal PQ duration values. These findings may contribute to the understanding of the process of cardiac electrical remodeling in athletes.
